# Transcriptomic profiling of human granulosa cells between women with advanced maternal age with different ovarian reserve

**DOI:** 10.1007/s10815-023-02915-8

**Published:** 2023-08-17

**Authors:** Zhi-cheng Jia, Yong-qian Li, Bo-wen Zhou, Qing-chang Xia, Pei-xuan Wang, Xiao-xuan Wang, Zhen-gao Sun, Ying Guo

**Affiliations:** 1 The First Clinical College, Shandong University of Traditional Chinese Medicine, Jinan, China; 2 College of Traditional Chinese Medicine, Shandong University of Traditional Chinese Medicine, Jinan, China; 3 Reproductive and Genetic Center of Integrative Medicine, Affiliated Hospital of Shandong University of Traditional Chinese Medicine, Jinan, China

**Keywords:** Transcriptomic profiling, Granulosa cells, Advanced maternal age, Diminished ovarian reserve, Ovarian aging, Ferroptosis

## Abstract

**Background:**

Age-related diminished ovarian reserve (DOR) is not absolute. Some advanced maternal age (AMA) still have normal ovarian reserve (NOR) and often show better pregnancy outcomes. Exploring the transcriptomic profile of granulosa cells (GCs) in AMA could lead to new ideas for mitigating age-related diminished ovarian reserve.

**Aim:**

This study aimed to analyze the transcriptomic profile of GCs in AMA with different ovarian reserve.

**Results:**

In total, 6273 statistically significant differential expression genes (DEGs) (|log2fc|> 1, *q* < 0.05) were screened from the two groups, among which 3436 genes were upregulated, and 2837 genes were downregulated in the DOR group. Through Gene Ontology (GO) and Kyoto Encyclopedia of Genes and Genomes (KEGG) pathway enrichment analysis, the potential functions of dysregulated genes in AMA with DOR or NOR were predicted. The GO enrichment analysis revealed that the DEGs were mainly enriched in obsolete oxidation–reduction process, mitochondrion, metal ion binding, ATP binding, etc. The KEGG pathway enrichment analysis revealed that the above-mentioned DEGs were mainly enriched in ferroptosis, regulation of actin cytoskeleton, oxidative phosphorylation, etc. Meanwhile, verification of the mRNA expression levels of DEGs revealed the possible involvement of “ferroptosis” in age-related diminished ovarian reserve.

**Conclusions:**

From a new clinical perspective, we presented the first data showing the transcriptomic profile in GCs between AMA with different ovarian reserve. At the same time, we identified the role of ferroptosis in the GCs of AMA, providing a new biological basis for studying ovarian aging and improving pregnancy outcomes of AMA.

## Introduction

Age alone has an effect on fertility. Women older than 35 are traditionally defined as women with advanced maternal age (AMA) [1]. According to the human biologic clock, female fertility starts at puberty and decreases after the age of 30, with a steep decrease after 35, culminating in the menopause at 50 years of age [2]. Decreased fertility in AMA is reflected in multiple aspects, including ovarian failure [3], decreased endometrial tolerance [4], and increased risk of complications during pregnancy [5]. As a reproductive organ, the ovary exhibits a rate of aging much faster than other somatic organs [6]. So, the diminished ovarian reserve (DOR) is one of the most significant reasons for AMA infertility.

A large number of studies have been conducted to confirm the complex regulatory mechanisms of age-related diminished ovarian reserve, including multi-omics [7] and follicular microenvironment dimensions [8]. To date, most studies on age-related diminished ovarian reserve focused on comparing differences between women of childbearing age and the AMA with DOR [9]. In clinical practice, the ovarian aging rate of some AMA is relatively slow, maintaining normal ovarian reserve (NOR) and often show better IVF and pregnancy outcomes, indicating that age-related ovarian reserve is not absolute. No studies have focused on the mechanisms of fertility differences between AMA with different ovarian reserve. The POSEIDON criteria, which provides a more detailed classification based on the ovarian response, divides the population into four groups [10]. Among all POSEIDON groups, group 4 subpopulations have been estimated to constitute 55% of patients. POSEIDON group 2 subpopulations are AMA with NOR, and its diagnostic criteria were as follows: (a) age ≥ 35 years (B) antral follicles count (AFC) > 5 or anti-Müllerian hormone (AMH) 1.2 ≥ ng/ml. POSEIDON group 4 subpopulations are AMA with DOR, and its diagnostic criteria were as follows: (a) age ≥ 35 years (B) AFC < 5 or AMH < 1.2 ng/ml. According to the POSEIDON criteria, this study explores the differences between DOR and NOR in AMA.

Granulosa cells (GCs), the highly specialized somatic cell that forms multi-layers surrounding the oocyte, are in close contact with the oocyte via transzonal projections and gap junctions. The quantity and quality of GCs are critical for oocyte nuclear and cytoplasmic maturation and for acquiring competency for fertilization and early stages of embryo development [11]. In the process of ovarian aging, mitochondrial dysfunction, apoptosis level [12], and oxidative stress level of GCs increase [13], which leads to aging GCs can cause oocyte dysfunction by blocking the meiosis process during oocyte maturation [14]. GCs are considered to be one of the best noninvasive approaches for evaluating oocyte quality and studying ovarian function [15]. In clinical practice, GCs are available biological samples to study female follicle development without wasting female oocytes, which is more ethical [16]. Transcriptomics is a discipline that studies the transcription of genes in cells and the regulation of transcription at a holistic level and is an essential tool for studying cellular phenotype and function [17]. Several investigators have successfully studied the GCs transcriptome to gain insight into the health of the oocyte. However, what has not yet been investigated is the transcriptomic profile of GCs in AMA with different ovarian reserve. With the modern tendency of delaying childbearing, it is necessary to pay attention to the fertility of AMA [18]. Exploring the transcriptomic profile of GCs in AMA could lead to new ideas for mitigating age-related diminished ovarian reserve.

## Material and methods

### Study population

Samples and clinical data were collected at the Center for Integrative Reproduction and Genetics, Affiliated Hospital of Shandong University of Traditional Chinese Medicine, from January 2022 to December 2022. Sixty patients were recruited in this prospective study.

In this study, we collected GCs derived from AMA (age ≥ 35 years) of two different ovarian reserve cohorts. Six samples from each group were selected randomly for sequencing analysis.

The study group (*n* = 30) comprised AMA with DOR. The diagnostic criteria adopted POSEIDON criteria: (a) 35 ≤ age ≤ 42 years (b) AFC < 5 or AMH < 1.2 ng/ml. The control group (*n* = 30) was AMA with NOR.

The exclusive criteria were uterine abnormalities, endometriosis and adenomyosis, diabetes, thyroid diseases, hyperprolactinemia, chromosomal abnormalities, repeated spontaneous abortion, and unexplained infertility. All participants had standard body mass index (BMI, ranged 18–28) and received IVF treatment with GnRH antagonist protocols. The study was authorized by the local institutional review board (Reproductive Ethics Committee of The Affiliated Hospital of Shandong University of Traditional Chinese Medicine, approval no. SDTCM/E2209-22). All patients provided written informed consent before sample collection.

### Controlled ovarian stimulation protocols and granulosa cells collection

All participants underwent IVF/ICSI treatment using a GnRH-ant protocol. Human chorionic gonadotropin (hCG, Lizhu, Zhuhai, China) or GnRH agonist (triptorelin acetate; France) combined hCG (dual trigger) was administered to trigger the maturation of oocytes. Oocyte pick-up (OPU) was performed by transvaginal ultrasound-guided needle aspiration 35–36 h following triggering, followed by standard IVF/ICSI as previously reported as previously described [19]. Follicular fluid aspirated from follicles of individuals during oocyte retrieval was pooled and considered independent samples. GCs were isolated from the follicular fluid by density gradient centrifugation. Follicular fluid was collected to a centrifuge tube for centrifugation at 380 × g for 5 min at 23 °C, and the supernatant was removed. Phosphate-buffered saline (5 mL) was added to the precipitate and mixed. Then, 5 mL of Ficoll-Paque (GE Healthcare, Chicago, IL, USA) was added to a 15-mL centrifuge tube. The suspension was added slowly to the upper layer of Ficoll-Paque, and centrifugation at 380 × g for 20 min at 23 °C was carried out. The GCs layer was aspirated and transferred to a 1.5-mL centrifuge tube, and repeated pipetting was undertaken followed by centrifugation at 380 × g for 3 min at 23 °C. The supernatant was discarded and GCs were identified using immunohistochemistry based on the expression levels of follicle stimulating hormone receptor and Müllerian inhibiting substance type II receptor. The final products were stored at −80 °C for later use. Women were totally informed of the procedures, and signed informed consent was obtained from all participants.

### RNA extraction and library construction

Total RNA was isolated and purified using TRIzol reagent (Invitrogen, Carlsbad, CA, USA), following the manufacturer’s protocol. Each sample’s RNA purity and concentration were quantified using NanoDrop ND-1000 (NanoDrop, Wilmington, DE, USA). The RNA integrity was assessed by Bioanalyzer 2100 (Agilent, CA, USA) with RIN number > 7.0, and confirmed by electrophoresis with denaturing agarose gel. Purified poly(A) RNA was obtained after two rounds of purification using Dynabeads Oligo (dT)25–61005 (Thermo Fisher, CA, USA) and fragmented into small pieces using Magnesium RNA Fragmentation Module (NEB, cat.e 6150, USA). The cleaved RNA fragments were reverse-transcribed to create the cDNA by SuperScript™ II Reverse Transcriptase (Invitrogen, cat. 1896649, USA), which were next used to synthesize U-labeled second-stranded DNAs with *E. coli* DNA polymerase I (NEB, cat.m0209, USA), RNase H (NEB, cat.m0297, USA), and dUTP solution (Thermo Fisher, cat.R0133, USA). A-bases were added to the blunt ends of each strand for ligation to the indexed adapters, which harbor T-base overhangs. A single- or dual-index adapters are ligated to the fragments, and size selection was performed with AMPure XP beads. After the heat-labile UDG enzyme (NEB, cat.m0280, USA) treatment of the U-labeled second-stranded DNAs, the ligated products are amplified with PCR. The average insert size for the final cDNA library was 300 ± 50 bp.

### Bioinformatics analysis of RNA-seq

We performed 2 × 150 bp paired-end sequencing (PE150) on an Illumina NovaSeq™ 6000 (LC-Bio Technology CO., Ltd., Hangzhou, China) following the manufacturer’s recommended protocol. Gene expression was represented by FPKM, and the expression of known genes in different samples was recorded. Differential expression analysis was conducted for genes that had been StringTie assembled and quantified using the R package edgeR; DEGs were defined as those with |log2 (fold change) |≥ 1 and *q* < 0.05. To understand the biological function of DEGs, GO term (http://geneontology.org) and KEGG pathway analysis (https://www.kegg.jp/kegg/) were implemented. A corrected *q* value < 0.05 was considered statistical significantly. In addition, we also selected a significantly enriched pathway—ferroptosis, mapped the heat map of the expression amount of related DEGs in transcriptome sequencing, and presented its KEGG pathway diagram to determine its role in age-related ovarian aging. The Search Tool for the Retrieval of Interacting Genes (STRING) database (ver. 11.5; https://string-db.org/) was used to elucidate the interactive relationships of the DEGs related to ferroptosis. The interacting pairs with a confidence score greater than 0.15 were considered significant and were retained. Subsequently, Cytoscape software (ver. 3.8.1) [20] was used to establish the protein–protein interaction (PPI) network.

### Quantitative reverse transcription PCR

To verify the confidence of RNA-seq, we selected several DEGs related to ferroptosis which were confirmed by RT-qPCR. Total RNA was isolated and purified using TRIzol reagent (Invitrogen, Carlsbad, CA, USA), following the manufacturer’s protocol. Each sample’s RNA purity and concentration were quantified using NanoDrop ND-1000 (NanoDrop, Wilmington, DE, USA). Total RNA was reverse-transcribed into cDNA using the TUREscript First Strand cDNA Synthesis Kit (Aidlab, Beijing, China) according to the manufacturer’s instructions. The primers (Table 1) were designed and synthesized by Beijing Tsingke Biotech Co., Ltd. (Beijing, China). qPCR was subsequently performed using an AceQ qPCR SYBR Green Master Mix system (Vazyme Biotech Co., Ltd.) according to the manufacturer’s instructions. The reaction protocol was as follows: initial denaturation at 95 °C for 3 min, denaturing at 95 °C for 10 s, and annealing at 60 °C for 30 s for 40 cycles. The relative expression levels of each gene were normalized according to glyceraldehyde 3-phosphate dehydrogenase (GAPDH) expression and analyzed using the 2-ΔΔCt method [21].

**Table 1:** The sequences of primers used for qRT-PCR analysis

Gene	Forward (sequence 5′–3′)	Reverse (sequence 5′–3′)
ACSL4	CCCTGAAGGATTTGAGATTCACA	CCTTAGGTCGGCCAGTAGAAC
FTH1	TCCTACGTTTACCTGTCCATGTC	GGTTCTGCAGCTTCATCAGTTTC
FTL	CAAGAATTCATGAGCTCCCAG	ACCCTCGAGTTAGTCGTGCTT
GPX4	AGAGATCAAAGAGTTCGCCG	TTGTCGATGAGGAACTGTGG
NCOA4	TGGGCCAGTTCAATTGTCTTACT	GCCATCAAGTGCTCAGGAATTTG
PRDX2	GACTCTCAGTTCACCCACCTG	CGTCCCACAGGCAAATCATTAAC
PRDX4	ACCCATCAGATCTCAAAGGACTATG	TGGAATGCTTGAACCAAACGTAG
PTGS2	CACAGTCTTCTCATCACTTCGTTTCTC	AAATAGCAGTCCTGAGCTGAGGTTTAC
TFRC	ACCTTTCGTCCCTGCATTTAAAG	GCCCAGTTGCTGTCCTGATATAG
GAPDH	GGAGCGAGATCCCTCCAAAAT	GGCTGTTGTCATACTTCTCATGG

### Statistical analysis

The K-S test was used for the normality test. Continuous variables are expressed as mean ± SD or median (IQR), and categorical variables are expressed as number (n) and percentage (%). Mann–Whitney *U* test or Student’s *t*-tests were used for continuous variables, and the Chi-square test was used for categorical variables. All statistical analyses were performed with the SPSS 25.0 statistical software (IBM, Chicago, IL, USA). A *P*-value < 0.05 was considered statistically significant.

## Result

### Baseline characteristics of the study population

Patients’ baseline characteristics are detailed in Table 2. There were no statistically significant differences in the mean age, types of infertility, duration of infertility, BMI, basal LH, E2, P4 level, stimulation duration of recombinant human follicle-stimulating hormone (rhFSH), total rhFSH, LH level on trigger day, and method of fertilization between the two groups (all *P* > 0.05). The basal FSH level was significantly higher in the DOR group than in the NOR group, while the AMH, AFC, E2 and P4 level on trigger day, number of oocytes retrieved, maturation oocytes, 2PN fertilization, available embryos, blastocyst, and frozen embryos were significantly lower than in the NOR group (all* P* < 0.05). There was no significant difference in the number of high-quality embryos and the proportion of high-quality embryos between the two groups (all *P* > 0.05).

**Table 2:** Comparison of general data between the two groups

	NOR (*n* = 30)	DOR (*n* = 30)	*P*
Age (years)	37.73 ± 2.03	38.13 ± 2.5	0.499
Types of infertility			0.152
Primary infertility	80.00%	63.30%	
Secondary infertility	20.00%	36.70%	
Infertility duration(years)	3.3 ± 2.47	4.07 ± 2.66	0.252
Gravidity (*n*)	1.6 ± 1.4	1.5 ± 1.59	0.797
Parity (*n*)	0.67 ± 0.71	0.5 ± 0.63	0.341
BMI (kg/m^2^)	23.58 ± 2.28	23.16 ± 2.63	0.515
AMH	2.79 ± 0.8	0.82 ± 0.27	0.00
Antral follicle count	12.1 ± 2.19	4.43 ± 0.57	0.00
Basal FSH level (IU/L)	5.99 ± 0.52	9.8 ± 1.56	0.00
Basal LH level (IU/L)	3.37 ± 1.62	4.12 ± 1.42	0.063
Basal E2 level (pg/ml)	39.45 ± 25.32	44.08 ± 18.92	0.425
Basal P4 level (ng/ml)	0.48 ± 0.31	0.54 ± 0.37	0.48
Stimulation duration of rhFSH (day)	8.53 ± 1.11	8.87 ± 1.55	0.341
Total rhFSH (IU)	2117.92 ± 530.96	2235.42 ± 582.21	0.417
LH level on trigger day (IU/L)	2.55 ± 1.8	3.31 ± 2.16	0.145
E2 level on trigger day (pg/ml)	1971.47 ± 1007.53	1157.86 ± 376.46	0.00
P4 level on trigger day (ng/ml	1.03 ± 0.39	0.76 ± 0.31	0.004
Method of fertilization			1.00
IVF	25/30(83.3%)	25/30(83.3%)	
ICSI	5/30(16.7%)	5/30(16.7%)	
Number of oocytes retrieved	10.27 ± 2.39	4.87 ± 1.25	0.00
Maturation oocytes	9.9 ± 2.71	4.57 ± 1.43	0.00
2PN Fertilization	6.6 ± 2.43	2.87 ± 1.48	0.00
Number of available embryos	3.37 ± 1.71	2.13 ± 1.28	0.002
Number of blastocyst	2.17 ± 2.38	0.4 ± 0.68	0.00
Number of high-quality embryos	1.2 ± 1.16	0.87 ± 1.14	0.265
Proportion of high-quality embryos	36/101(35.6%)	26/64(40.6%)	0.52
Number of frozen embryos	1.8 ± 1.77	0.9 ± 1.16	0.023

### Transcriptional profiles

We calculated the Pearson correlation coefficient of every two samples using gene expression.

**Fig. 1:**
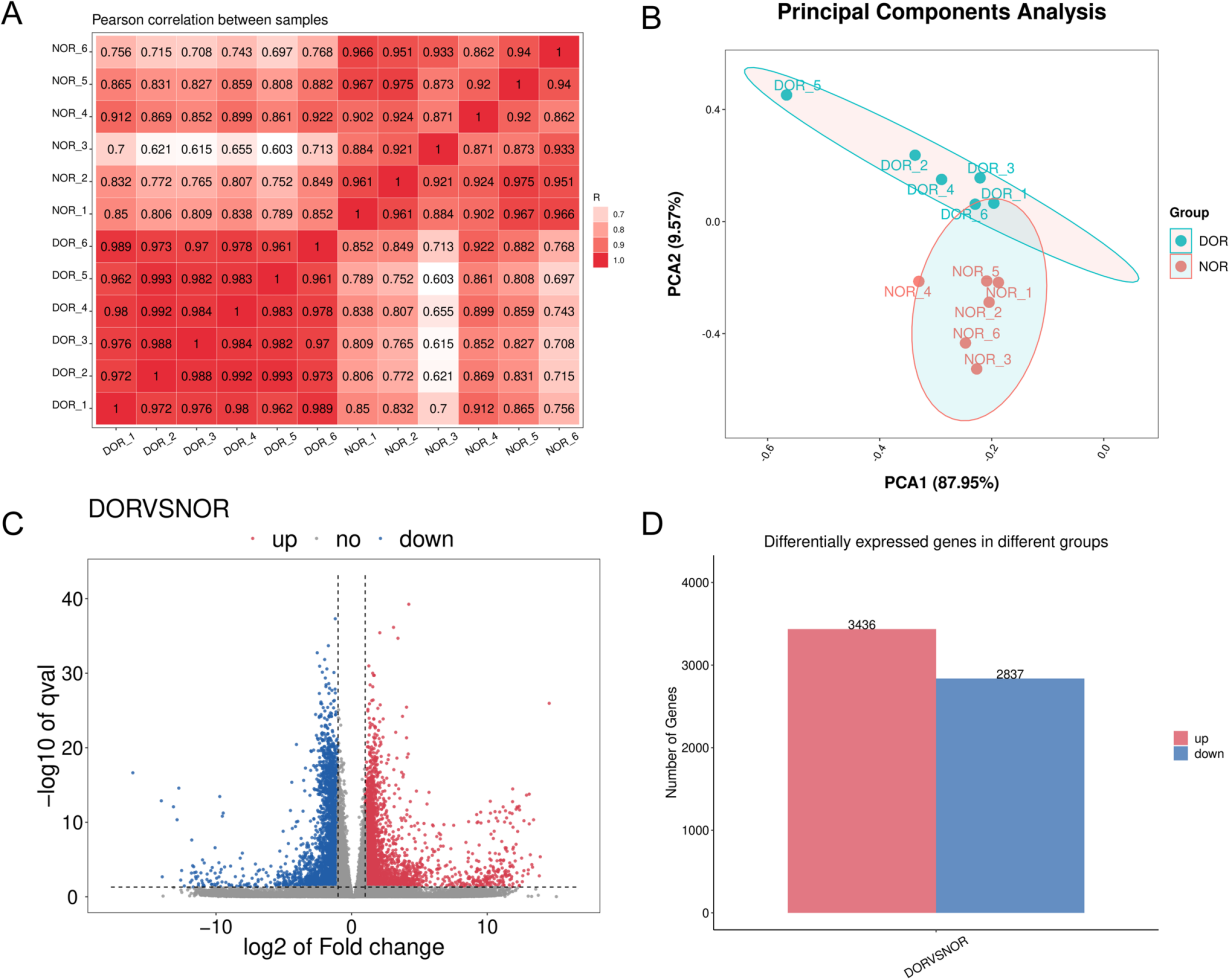
Differential expression analysis in DOR and NOR. **A** Pearson correlation coefficient between two groups of granulosa cells; **B** unsupervised PCA of two groups of granulosa cells (6 DORs and 6 NORs); **C** volcano plot shows DEGs in DOR and NOR; **D** bar chart showed the number of upregulated and downregulated DEGs in DOR compared with NOR

The expression patterns were generally homogeneous between every two samples. There are significant differences in the expression patterns of each sample between the two groups, while the gene expression patterns within the group are homogeneous, all R^2^ > 0.9 except for NOR_3 (Fig. 1A). The principal components analysis (PCA) showed that the DOR groups exhibited distinct gene expression profiles compared with NOR groups (Fig. 1B). In total, 6273 statistically significant DEGs (|log2fc|> 1, *q* < 0.05) were screened from the two groups, among which 3436 genes were upregulated, and 2837 genes were downregulated in the DOR group (Fig. 1C).

### GO and KEGG enrichment analysis

**Fig. 2:**
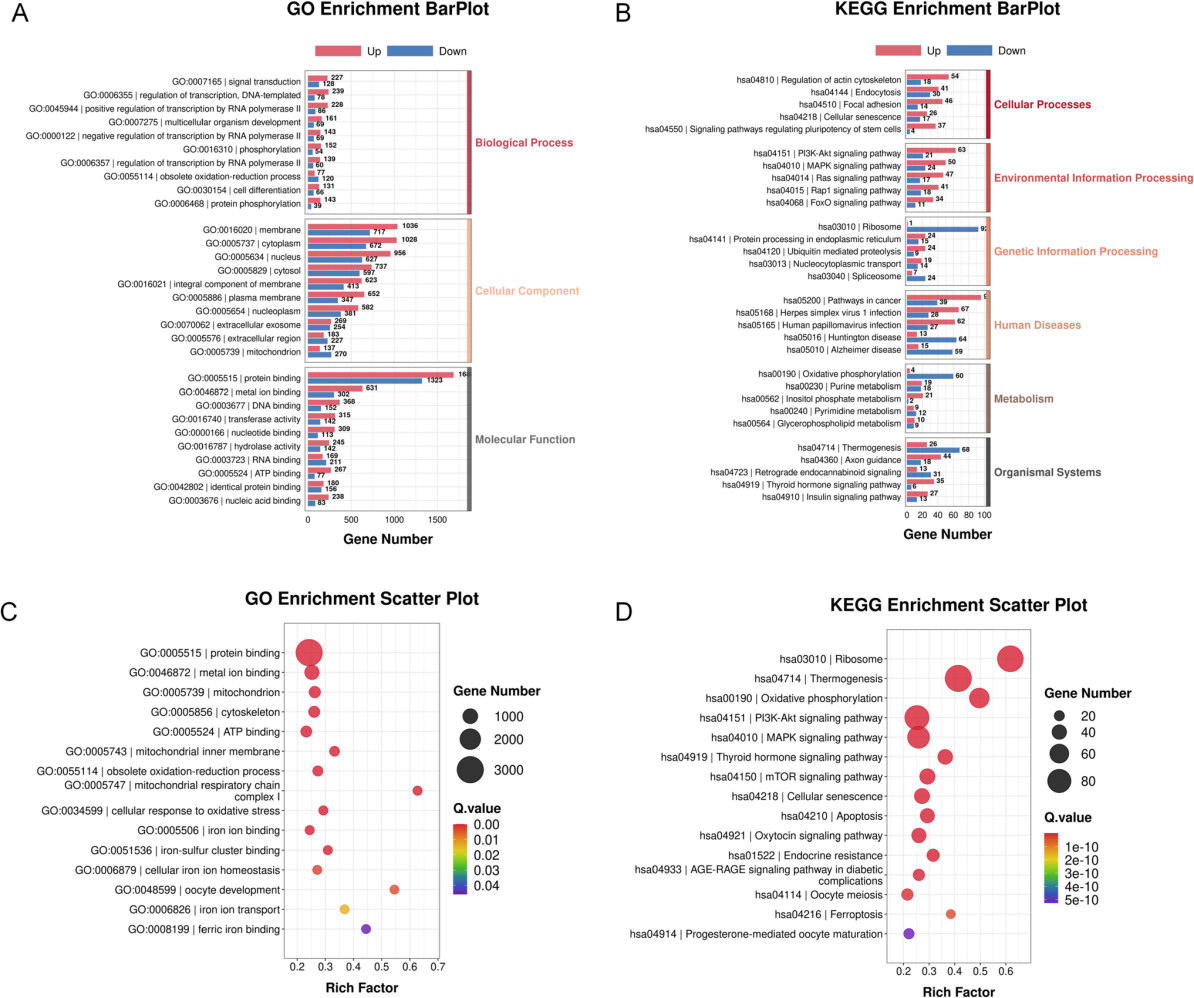
GO and KEGG enrichment analysis. **A** GO Bar plot; **B** KEGG bar plot; **C** GO scatter plot; **D** KEGG scatter plot

GO and KEGG pathway analyses were performed to determine the possible functions of 6273 DEGs. The GO enrichment analysis was performed to explore the biological functions of DEGs, and three categories of biological functions were identified, including biological process, cellular component, and molecular function. The top ten representative GO enrichment biological processes in each category are shown in Fig. 2A. Signal transduction, membrane, and protein binding were the most enriched biological process, cellular component, and molecular function, respectively. Meanwhile, the KEGG analysis divides biological metabolic pathways into six categories: cellular processes, environmental information processing, genetic information processing, human diseases, metabolism, and organismal systems. The top five representative KEGG enrichment results in each category are shown in Fig. 2B. GO enrichment scatter plot and KEGG enrichment scatter plot are shown in Fig. 2C and D, among which are some associated with the oocyte development, including the obsolete oxidation–reduction process, oxidative phosphorylation, endocrine resistance, and oocyte meiosis.

### Ferroptosis-related DEGs and pathview

**Fig. 3:**
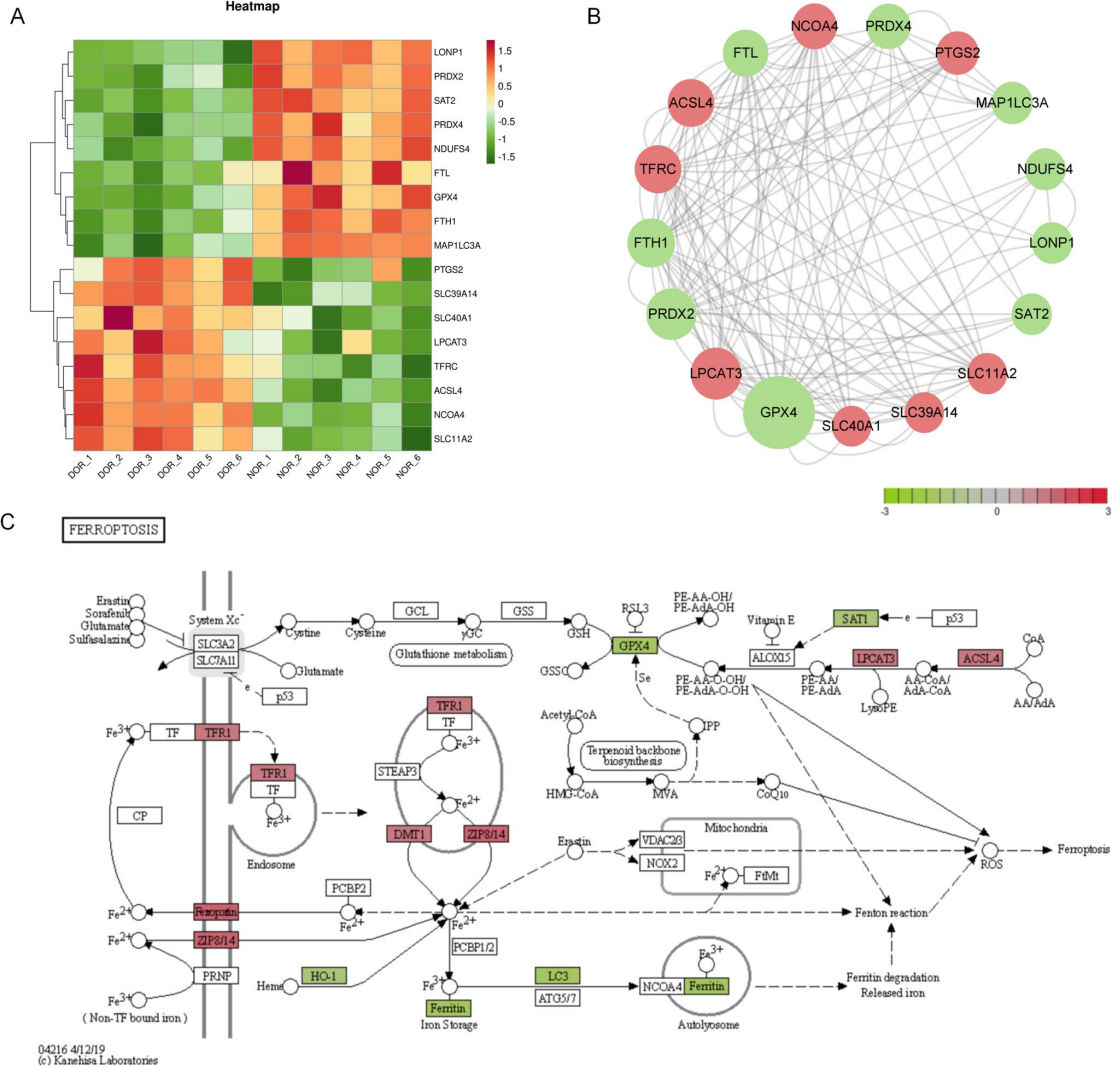
Ferroptosis related DEGs and pathview. **A** Heatmap showing DEGs related to ferroptosis; **B** PPI networks of DEGs related to ferroptosis; **C** pathview plot showing DEGs related to ferroptosis. Gene expression levels are indicated as significantly higher (red) or lower (green) in DOR compared to NOR

We examined the RNA sequencing dataset for the presence of gene transcripts correlative to ferroptosis in GCs. We presented a heatmap related to ferroptosis according to DEGS (Fig. 3A). The network topology attributes indicators of DEGs related to ferroptosis were analyzed using CytoNCA in Cytoscape software, including degree centrality, betweenness centrality, and closeness centrality. A PPI diagram was drawn based on the betweenness centrality, showing that glutathione peroxidase 4 (GPX4) is one of the most important hub nodes (Fig. 3B). Meanwhile, ferroptosis is significantly enriched in KEGG, and the pathview plot shows significant changes in ferroptosis markers such as GPX4, ferritin heavy chain 1 (FTH1), and acyl-CoA synthetase long-chain family member 4 (ACSL4) (Fig. 3C).

### Verification of the mRNA expression levels of DEGs

**Fig. 4:**
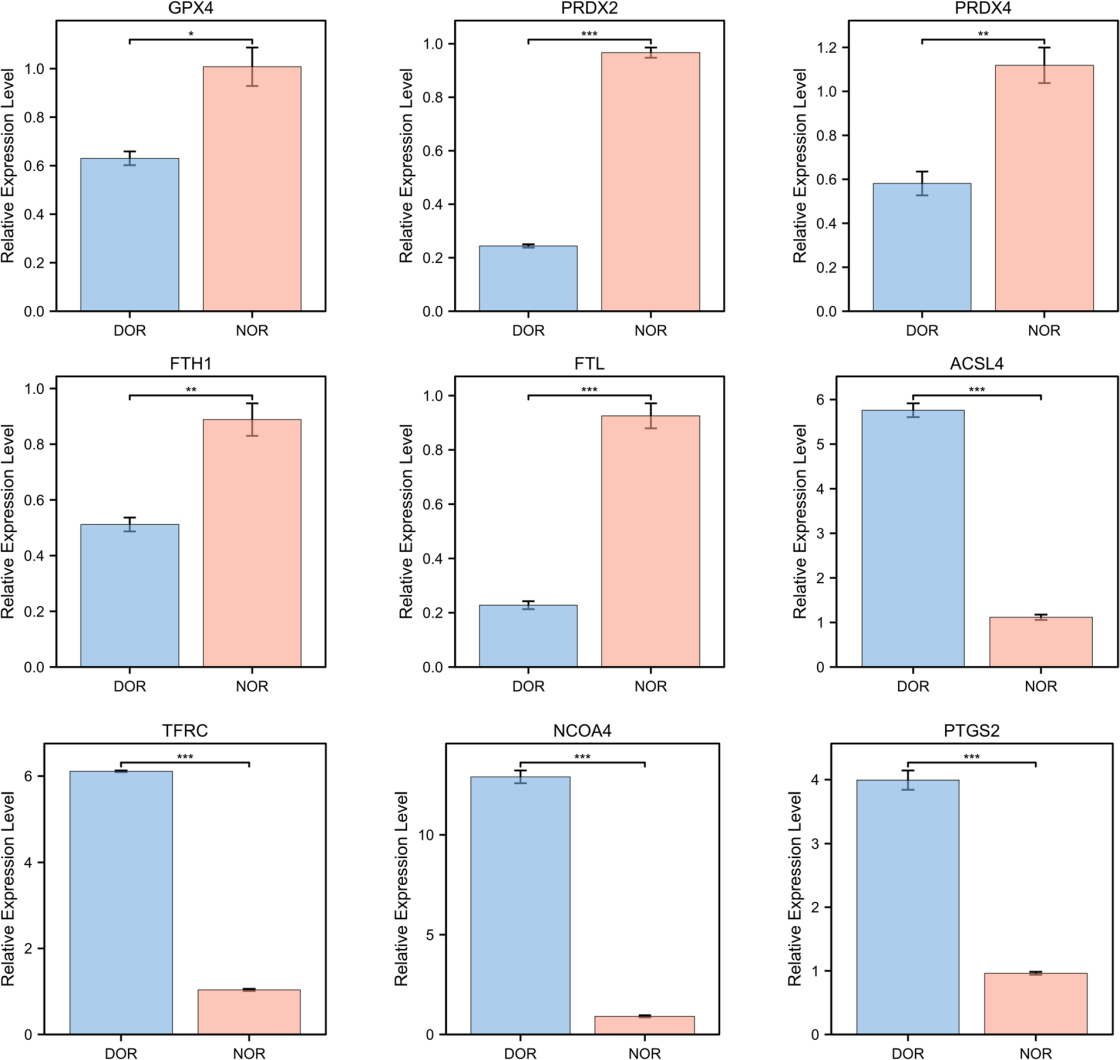
Verification of the expression levels of DEGs by qRT-PCR. *P < 0.05, *P < 0.01, *P < 0.001, DOR vs. NOR

Based on the sequencing data and functional analyses, nine DEGs related to ferroptosis were confirmed by RT-qPCR. Among the nine genes, five genes (GPX4, FTH1, FTL, PRDX2, PRDX4) exhibited by RNA sequencing in the DOR group were confirmed downregulation, and four genes (TFRC, ACSL4, PTGS2, NCOA4) were confirmed upregulation (Fig. 4).

## Discussion

So far, no studies have revealed the transcriptomic profile between AMA with different ovarian reserve. In this trial, we recruited AMA with different ovarian reserve and collected GCs for transcriptomic sequencing, showed the transcriptome landscape of GCs between AMA with different ovarian reserve for the first time, and verified the expression level of ferroptosis-related genes.

In clinical practice, the ovarian reserve of AMA varies from person to person. We prospectively collected GCs from AMA and tried to control variables as much as possible, such as using GnRH-ant protocol for all participants. Clinical data shows no difference in the dosage and duration of rhFSH medication between the two groups. However, the IVF outcomes of the DOR group were not as optimistic as those of the NOR group, including the number of oocytes retrieved and embryological outcomes. The unsatisfactory number of available embryos, blastocyst, and frozen embryos in the DOR group is highly likely due to limitations in the number of retrieved oocytes [22]. Previous studies have shown that AMA recruits fewer oocytes but has better quality compared to young women [23]. In our study, all patients were AMA, excluding young women, and there was no significant difference in the number and proportion of high-quality embryos between the two groups. This may be due to sample size limitations, or it may be the case that age has a deeper impact on the quality of oocytes, regardless of the number of oocytes, which requires larger sample studies to verify. In summary, it is undeniable that the IVF outcomes in the DOR group are worse than those of the NOR group. In clinical practice, the AMA with DOR is the most pessimistic population. Immediate IVF may be considered a first-line treatment strategy in women older than 38 to 40 years [24]. Further research is needed on controlled ovarian stimulation protocol and embryo transfer strategy for AMA.

The transcriptome landscape of the two groups of GCs showed significant differences in the expression patterns of each sample between the two groups. In contrast, the gene expression patterns within the group are homogeneous. A large amount of DEGs also indicates the differences in biological regulation processes between GCs behind the differences in clinical phenotypes between the two groups. Through GO and KEGG pathway enrichment analysis, the potential functions of dysregulated genes in AMA with DOR or NOR were predicted. The GO enrichment analysis revealed that the DEGs in AMA with different ovarian reserve were mainly enriched in obsolete oxidation–reduction process, mitochondrion, metal ion binding, ATP binding, etc. Meanwhile, the KEGG pathway enrichment analysis revealed that the above-mentioned DEGs were mainly enriched in ferroptosis, regulation of actin cytoskeleton, cellular senescence, PI3K-Akt signaling pathway, MAPK signaling pathway, oxidative phosphorylation, etc. Previous studies have shown significant differences in the transcriptome profile of GCs between AMA and young women and confirmed the dynamic nature of mitochondrial biogenesis [25, 26] and oxidative stress [27] in GCs in the ovarian aging process. With the increase in the women’s age, the activity of the antioxidant system decreases, leading to the continuous accumulation of reactive oxygen species, which together leads to an increase in oxidative stress damage in the ovaries [28]. Exploring oxidative stress-related processes might be a promising strategy against ovarian aging [29]. Mitochondria, as a tool for oocyte regeneration, are one of the most potential targets for alleviating ovarian aging [30]. In addition, both PI3K-Akt and MAPK signaling pathway are important pathways for primordial follicle activation, oocyte quality, and aging [31, 32]. The overlap between our transcriptome profiling and previous studies reflects the role of these classic pathways in ovarian aging and the reliability of our data.

Ferroptosis, an iron-dependent form of non-apoptotic cell death in 2012, has seen exponential growth in research over the past few years [33]. Ferroptosis has also made certain research progress in female reproduction, including its role in ovarian cancer [34], endometriosis [35], and polycystic ovary syndrome [36]. Ferroptosis is closely related to ovarian reserve and is a crucial pathway activated within immature ovarian follicles [37]. The upregulation of transferrin and ferritin in the ovary causes iron accumulation, induces ferroptosis, and inhibits estradiol biosynthesis [38]. Basonuclin1 is a mutated gene from the Chinese primary ovarian insufficiency lineage, and its deficiency triggers oocyte ferroptosis through the NF2-YAP pathway [39]. At the same time, ferroptosis inhibitor ferrostatin-1 can protect ovarian function by reducing GCs injury by regulating DNA methylation [40]. However, the ferroptosis in age-related ovarian aging has not been elucidated. GPX4, at the crossroads of lipid homeostasis and ferroptosis, the functional inactivation of GPX4 results in an increased lipid-ROS accumulation and subsequent lipid peroxidation [41]. Meanwhile, ACSl4 promotes sensitivity to ferroptosis by shaping cellular lipid components [42]. The GPX4 is an important factor in ovarian ultrastructural changes [43] and a potential target for treating ovarian clear cell carcinoma [44]. Peroxiredoxin 2 (PRDX2) and peroxiredoxin 4 (PRDX4), the member of the PRDX family, are vital antioxidants in cells. Knockdown of PRDX2 and PRDX4 can elevate cellular reactive oxygen species levels and induce ferroptosis [45]. PRDX2 inhibits ferroptosis via regulation of the GPX4/ACSL4 axis [46], and its role in age-related ovarian aging in mice has also been confirmed [47]. Prostaglandin-endoperoxide synthase 2 (PTGS2) also plays an important role in oocyte and embryonic development, and its upregulation is a result of GPX4 loss [48]. It is also recognized as a biomarker of lipid peroxidation occurring along with ferroptosis [49]. Ferroptosis is closely related to iron metabolism, and iron uptake, storage, utilization, and efflux are the main links of iron metabolism. Transferrin receptor (TFRC) is a universal iron importer for all cells using extracellular transferrin [49]. FTH1 and FTL (ferritin light chain) are the main iron storage proteins. The increase of TFRC and the decrease of FTL and FTH1 indicate that iron uptake increases and iron storage decrease, suggesting iron overload during ferroptosis. Nuclear receptor coactivator 4(NCOA4), a cargo receptor for ferritinophagy, directly recognizes and binds FTH1 [50]. Disrupting NCOA4-FTH1 interaction can inhibit ferritinophagy and ferroptosis [51]. The genes mentioned above were differentially expressed in the sequencing data, and the verification of the mRNA expression also confirmed their expression trend in GCs, confirming the role of ferroptosis in GCs of AMA with different ovarian reserve.

Our research is limited to the study of the transcriptome landscape of GCs, which is an exploratory study. Due to ethical restrictions, we have not conducted oocyte and embryo level research. The conclusions cannot prove that these genes are involved in oocyte aging and embryo development. Due to funding and sample size limitations, we have not recruited more subgroups, such as the differences and connections between young women with different ovarian reserve and AMA. We will conduct in-depth research based on the existing foundation.

## Conclusions

In conclusion, this study revealed the transcriptomic profile of GCs in AMA with different ovarian reserve for the first time and reported the relationship between ovarian aging and ferroptosis. From a new clinical perspective, it provides a new biological basis for studying ovarian aging and improving pregnancy outcomes of AMA.

## Data Availability

The transcriptomic data was registered in the GEO database of the NCBI (http://www.ncbi.nlm.nih.gov/geo/) with the accession number GSE232306.
